# Natural Norovirus Infections in Rhesus Macaques

**DOI:** 10.3201/eid2207.151740

**Published:** 2016-07

**Authors:** Tibor Farkas

**Affiliations:** Cincinnati Children’s Hospital Medical Center, Cincinnati, Ohio, USA

**Keywords:** enteric infections, noroviruses, Norwalk virus, primates, rhesus macaques, USA, viruses, zoonoses

## Abstract

Using a recently developed real-time reverse transcription PCR, I retested 500 fecal samples from rhesus macaques collected in 2008. Previous conventional reverse transcription PCR testing identified 1 isolate of GII norovirus; retesting found GI, GII, and possible GIV noroviruses in the samples, indicating the natural circulation of noroviruses in nonhuman primate colonies.

Noroviruses are a leading cause of acute gastroenteritis in humans and have also been described in several animal species. Although the pathologic role of most animal noroviruses is not clearly established, evidence of the close genetic relatedness of animal and human noroviruses and the detection of animal norovirus–specific antibodies in humans and of human norovirus specific–antibodies in different animal species strongly suggest zoonotic or interspecies transmission of noroviruses (*1*–*4*). However, zoonotic norovirus infections have not yet been reported, perhaps because zoonotic transmission of noroviruses is rare or the routine norovirus detection techniques are not designed to detect animal strains. On the other hand, experimental infection of several animal species has clearly shown that human norovirus strains are able to replicate in animals, including gnotobiotic pigs and calves and nonhuman primates (*5**–**8*). In addition, molecular detection of GII.12 and GII.4 noroviruses, which are usually human strains, has been reported in swine, cattle, and pet dogs (*9**,**10*).

## The Study

A previous study evaluated samples that were collected during 2008 from the rhesus macaque (*Macaca mulatta*) colony housed at the Tulane National Primate Research Center (Covington, LA, USA) to determine the presence of caliciviruses (*11*). Using a broadly reactive primer pair (P289/P290), this study detected diverse recovirus strains and 1 GII norovirus (FT244, GenBank accession no. HM035148) in 1 macaque (*11*). Recently, a quantitative real-time reverse transcription PCR (RT-PCR) for the detection of GI, GII, and GIV noroviruses was developed (*12*). In 2015, I used this highly specific and sensitive assay to retest the 500 rhesus macaque fecal samples from the previous study, including the sample that contained FT244, to determine the presence of noroviruses.

**Figure 1:**
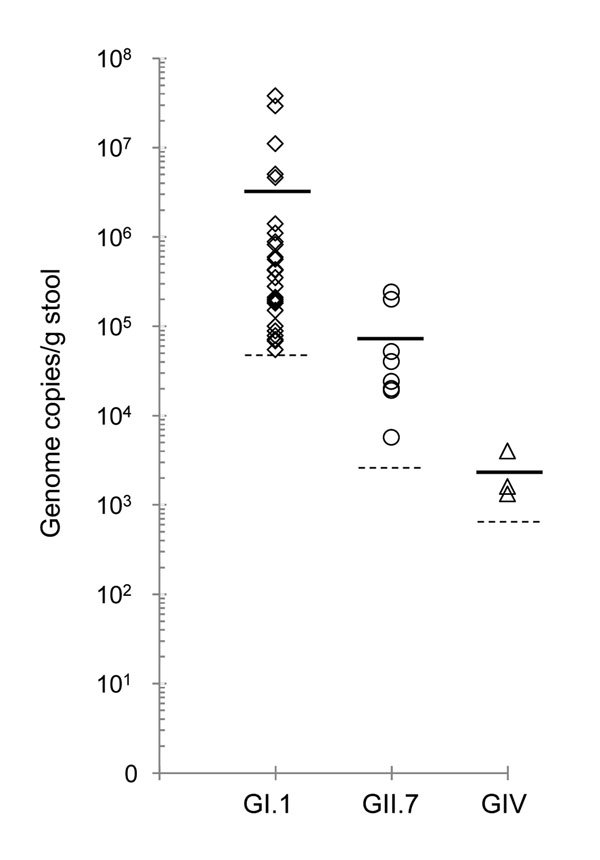
Norovirus genome copies per gram of rhesus macaque fecal samples collected in 2008 (*11*) and retested in 2015 by using a highly sensitive and specific real-time reverse transcription PCR. Shown are samples positive for GI (n = 30; diamonds), GII (n = 8; circles), and GIV (n = 3; triangles) noroviruses. Solid lines represent mean viral load; dashed lines represent the corresponding detection limits of the multiplex assay.

Of the 500 samples, 41 (8.2%) showed a cycle threshold (C_t_) lower than the assay’s cutoff value. These positive samples included 30 (6%) GI (C_t_ 29.60–39.98), 8 (1.6%) GII (C_t_ 32.96–38.76), and 3 (0.6%) GIV (C_t_ 38.30–39.88) noroviruses. The viral load in the GI (mean 3.2 × 10^6^ copies/g) and GII (mean 7.5 × 10^4^ copies/g) norovirus-positive samples were in the range reported for human fecal samples (*13*). All GIV-positive samples had viral loads close to the assay’s detection limit (1.3 × 10^3^ copies/g) (Figure 1). The sample that was positive for the GII norovirus (FT244) in the previous study had a viral load of 2.0 × 10^5^ genome copies/g. Further analysis by sequencing of region D amplicons (*14*) revealed that the GI-positive samples contained GI.1 noroviruses and the GII-positive samples contained GII.7 noroviruses. However, I could not obtain RT-PCR products and sequence information for the GIV-positive samples, even by nested RT-PCR. Consequently, I concluded that these samples either gave a false-positive result (C_t_ values close to the detection limit) by real-time RT-PCR or contained a novel GIV norovirus that could not be amplified efficiently by primers designed on the basis of currently available GIV norovirus sequences. 

**Figure 2:**
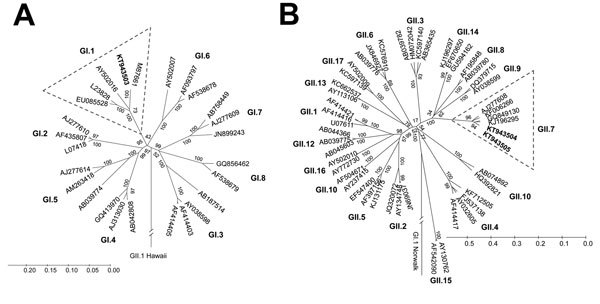
Phylogenetic trees showing GI and GII noroviruses from rhesus macaque fecal samples collected in 2008 (*11*) and retested in 2015 by using a highly sensitive and specific real-time reverse transcription PCR. GenBank accession numbers or other isolate identifiers are shown. Bold indicates isolates detected in this study. A) GI noroviruses share 100% nucleotide homology with the prototype Norwalk virus GI.1 strain (M87661). B) GII noroviruses group with GII.7 human noroviruses. Three of the 4 GII norovirus open reading frame (ORF) 2 sequences obtained in this study were identical. Only nonidentical sequences are shown. Phylogenetic trees were constructed on the basis of alignments of full length ORF2 nucleotide sequences, by using the unweighted pair group method with arithmetic mean and the neighbor-joining clustering methods of the Molecular Evolutionary Genetics Analysis (MEGA version 6.1; http://mega.software.informer.com/6.1/) software with Jukes-Cantor distance calculations. The confidence values of the internal nodes were obtained by performing 1,000 bootstrap analyses. Scale bars represent nucleotide substitutions per site.

In addition, I obtained full-length open reading frame (ORF) 2 sequences from 2 GI-positive and 4 GII-positive samples. The GI.1 norovirus ORF2 sequences (1,593 nt) had 100% nucleotide homology with each other and with the prototype Norwalk virus (M87661) (Figure 2, panel A). The GII.7 norovirus ORF2 sequences (1,623 nt) exhibited 99%–100% nucleotide homology with each other and 95% nucleotide homology with the closest GII.7 strain in the GenBank database (accession no. KJ196295) (Figure 2, panel B). I deposited 3 ORF2 sequences obtained in this study into the GenBank database under accession nos. KT943503–KT943505.

## Conclusions

A previous study reported the molecular detection of a GII norovirus in 1 of 500 rhesus macaques tested (*11*). Although this detection rate was extremely low (0.2%), the finding indicated the occurrence of natural norovirus infections in colony macaques. In this study, retesting the 500 samples by a more sensitive and specific real-time RT-PCR confirmed the previously detected presence of the GII norovirus in 1 sample but also identified additional samples positive for GI, GII, and GIV noroviruses. Furthermore, additional amplification of the viral genome and sequencing confirmed the presence of GI.1 and GII.7 noroviruses but not GIV noroviruses.

The detection of a GI.1 norovirus in samples collected in 2008, with 100% homology to the prototype Norwalk virus, is somewhat surprising. The prototype virus was originally described in an outbreak occurring during 1968 (*15*). According to outbreak surveillance data, GI.1 norovirus infections are extremely rare; consequently, establishing whether the Norwalk strain is still in circulation or has completely disappeared is difficult. Data on human norovirus strains circulating in the community or present in environmental samples at the time of the rhesus macaque fecal sample collection were not available from the local health department. The fecal samples were collected by rectal loops from individual macaques, so environmental contamination of samples, which might be an issue in other studies where manure is collected (*9*), can be ruled out. Samples positive for Norwalk virus were not present in the laboratories involved in the study, and rhesus macaque samples were stored in a separate freezer under Biosafety Level 2+ protocol. This finding is supported by a report of recent circulation of noroviruses with 100% nt homology to the Norwalk virus (GenBank accession nos. JX455860, JX455863, JX455870, and JX455872) that were found in sewage samples in Tunisia during 2007–2009.

The observations from this study and the previous study (*11*) indicate a strong plausibility for nonhuman primate reservoirs of human norovirus infections and the genetic mixing of animal and human caliciviruses under natural conditions from which new strains or emerging pathogens may arise. Additional studies are needed to establish the frequency, identity, and relevance of norovirus infections in nonhuman primates.
